# Streaming Potential with Ideally Polarizable Electron-Conducting Substrates

**DOI:** 10.1021/acs.langmuir.2c01305

**Published:** 2022-08-04

**Authors:** Andriy Yaroshchuk, Emiliy Zholkovskiy

**Affiliations:** † ICREA, Pg. Lluís Companys 23, 08010 Barcelona, Spain; ‡ Department of Chemical Engineering, Universitat Politècnica de Catalunya - BarcelonaTech, Av. Diagonal 647, 08028 Barcelona, Spain; § F. D. Ovcharenko Institute of Bio-Colloid Chemistry, National Academy of Sciences of Ukraine, Vernadskiy Blvd. 42, 03142 Kyiv, Ukraine

## Abstract

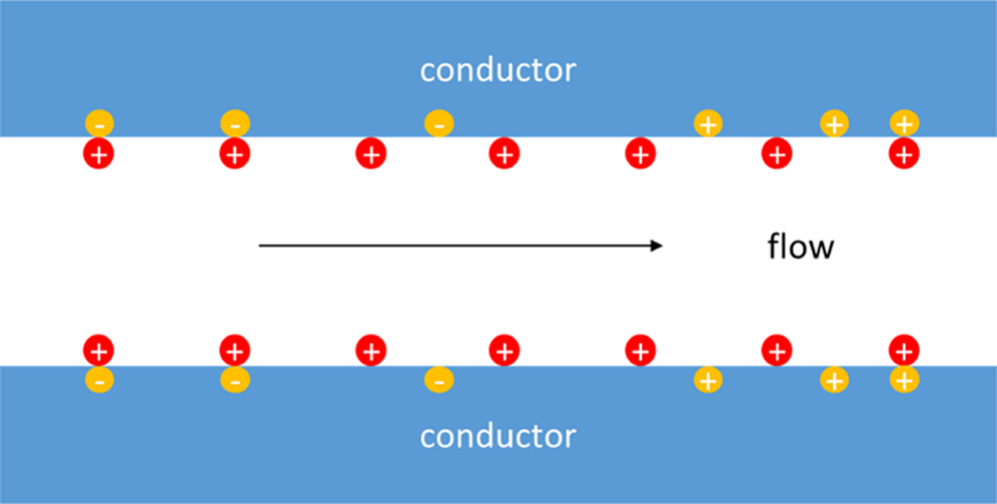

With nonconducting substrates, streaming potential in sufficiently broad (vs Debye screening length) capillaries is well known to be a linear function of applied pressure (and coordinate along the capillary). This study for the first time explores streaming potential with ideally polarizable electron-conducting substrates and shows it to be a nonlinear function of both coordinate and applied pressure. Experimental manifestations can be primarily expected for streaming potentials arising along thin porous electron-conducting films experiencing solvent evaporation from the film side surface. Model predictions are in good qualitative agreement with literature experimental data.

## Introduction

Foundations of the classical theory of streaming potential were laid down more than a century ago.^1^ The initial and consequent models have considered non-electron-conducting substrates.^2^ Several studies considered ion-conducting, namely, porous substrates (see, for example, refs (3−5)) but the physics in this case is essentially different due to the relatively low conductivity of such substrates and lack of ideal polarizability of interfaces between them and electrolyte solutions. Recently, a new interesting context for electrokinetic phenomena of streaming potential (and streaming current) has arisen in capillarity-driven energy harvesting from evaporation with (nano)porous materials (see, for example, refs (6−9); the state of the art of this emerging field has very recently been critically reviewed in ref (10)). In this case, hydrostatic pressure drops can be very high being ultimately controlled by capillary pressures in nanopores. At the same time, several relevant experimental studies used electron-/hole-conducting nanoporous substrates.^8,9,11^ Below, we will see that the combination of large hydrostatic pressure drops with solid-substrate electron conductance can make steaming potential essentially different from the classical case.

Some transport phenomena (membrane potential, electrical conductance, and pressure-driven salt rejection) in electrolyte-filled nanopores with electron-conducting walls have been recently explored by Ryzhkov et al.^12−17^ These phenomena are nontrivial only when there is a noticeable overlap of diffuse parts of electric double layers (EDLs). Such systems afford only numerical analysis. Besides, principal emphasis was made on the impact of an external bias while the role of redistribution of electron charges in floating (ungrounded) systems was less explored. Electrokinetic phenomena were not considered.

In this study, for the first time, we account for electron/hole conductance of matrices of porous materials experiencing flow-induced streaming potential. To obtain simple analytical results, we consider the limiting case of sufficiently broad capillaries without any appreciable overlap of diffuse parts of EDLs and surface conductance phenomena as well as neglect the existence of the so-called Stern layer.^18^ The existence of the latter may be important in more concentrated solutions and close to strongly charged surfaces. Below, we will see that strong surface charges (chemical plus induced) may well arise close to the channel exit under strongly nonlinear conditions. Therefore, accounting for the Stern layer is an essential next step that will be made in future studies.

We also discuss scenarios of possible experimental manifestations and demonstrate that they can be expected for rather large hydrostatic pressure differences and in sufficiently dilute electrolyte solutions. In combination with the requirement of negligible EDL overlap (implying relatively large pore size), this may be difficult to achieve in pressure-driven processes. However, we will see that situation can be different in systems where large hydrostatic pressure gradients are induced by capillarity in water evaporation from hydrophilic nanopores.

## Theory

**Figure 1:**
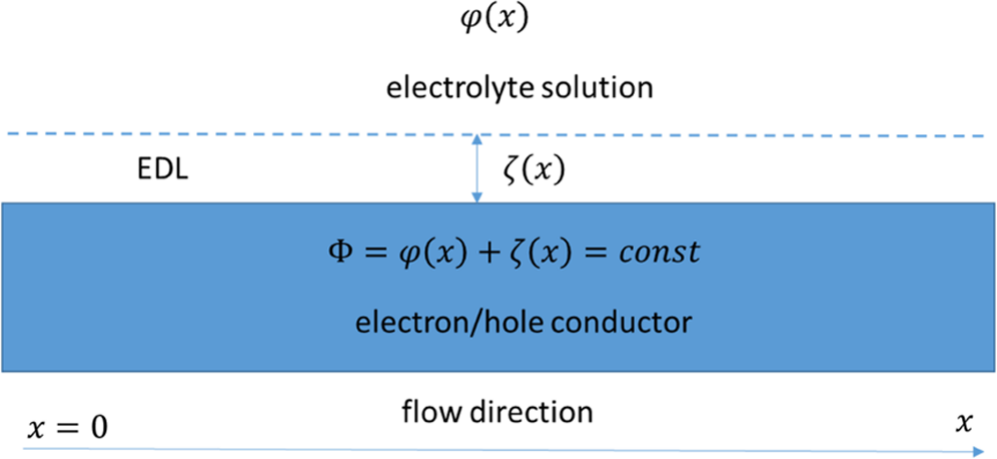
Schematic and system of coordinates.

Streaming currents arise as a result of the advective movement of electrically charged liquids close to “charged” solid/liquid interfaces in electrolyte solutions. Strictly speaking, the total electric charge of the interface region is zero; however, a charge is “bound” to the surface while its “counter-charge” can move with and/or relative to the liquid. Advective movement of electrolyte solution through a capillary with “charged” walls gives rise to a convective current. In streaming potential mode, external circuit is open, so the net electric current must be zero in any capillary cross section. Streaming potential is the voltage arising to compensate exactly the convective streaming current by an electromigration current in the opposite direction. The local density of convective current is equal to the product of local electric charge density and fluid velocity. Expressing the space-charge distribution via electrostatic potential by the Poisson equation, using the Stokes equation with the standard boundary condition of no slip on the capillary wall and taking into account the zero-current condition, for sufficiently broad (compared to the Debye screening length) capillaries, one can obtain this celebrated Smoluchowski formula^2^
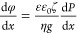
1where φ is the electrostatic potential in the central part of the capillary (far away from its walls), ζ is the potential drop within diffuse parts of EDLs occurring at the surface (the so-called ζ-potential), εε_0_ is the fluid dielectric constant, η is the fluid viscosity, *g* is the (bulk) electrical conductivity of electrolyte solution. The potentials and the system of coordinate are schematically shown in Figure 1. With electron-conducting substrates, the electrostatic potential of the conductor surface must be the same all the way along the capillary. Let us denote its constant value by Φ
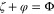
2

By substituting eq 2 into eq 1, we obtain
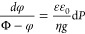
3

In this simple analysis, we neglect the influence of electrokinetic phenomena on the volume flow (this is justified in sufficiently broad capillaries). Therefore, the hydrostatic pressure is independent of electrostatic potential and its profile is linear. Accordingly, eq 3 can be easily integrated along the capillary to yield
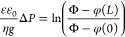
4where *L* is the channel length
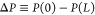
5is the hydrostatic-pressure difference along the capillary. From eqs 4 and 5, we obtain
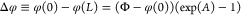
6
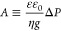
7

The hydrostatic pressure profile is linear
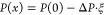
8where ξ ≡ *x*/*L* is the dimensionless coordinate along the channel. Taking this into account, from eq 4, we obtain
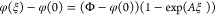
9

In contrast to the classical case of dielectric substrates, the electrostatic potential profile is nonlinear. The extent of nonlinearity is controlled by parameter *A*.

This analysis assumes that there are some fixed charges on the capillary walls (sometimes referred to as “chemical charge”) arising due to preferential ion adsorption or dissociation of ionogenic groups. Therefore, there is a nonzero ζ-potential at zero volume flow. Under flow conditions, additional electrostatic potential arises outside EDLs and at the capillary walls owing to the appearance of net electric charges at the capillary edges. Physically, the constancy of surface electrostatic potential is ensured by the appearance of polarization electron/hole charges at the capillary surface. Together with the initially present “chemical” charges, these polarization charges give rise to a position-dependent ζ-potential that can be found from the condition of constancy of full surface electrostatic potential (eq 2) and distribution of electrostatic potential outside the EDLs (eq 9)
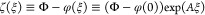
10

We consider the conductor ungrounded. Therefore, the total induced electron/hole charge must be zero. There is this well-known relationship between surface-charge density and equilibrium electrostatic potential at a charged surface^2^
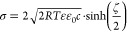
11so surface-charge density is proportional to the hyperbolic sinus of ζ-potential. For simplicity, let us initially assume that the “chemical” charge remains unchanged under flow conditions (constant charge approximation). Taking into account this and the fact that the total surface charge under flow conditions must be equal to the “chemical” charge, we obtain
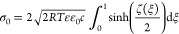
12where  is the (coordinate-independent) density of “chemical” surface charge and ζ_0_ is the ζ-potential under no-flow conditions. Equation 12 can be rewritten as
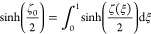
13

Substituting eq 10 for the distribution of ζ-potential, we obtain
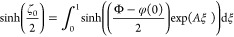
14

The integral in the right-hand side of eq 14 can be taken to yield
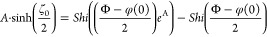
15where *Shi* is the integral hyperbolic sinus. Equation 15 is a transcendental equation for the determination of (Φ – *φ*(0)) as a function of parameter *A*, which is proportional to the hydrostatic pressure drop along the channel. Φ and *φ*(0) enter eq 15 only in combination, (Φ – *φ*(0)), so they cannot be determined separately. However, the pressure dependence of streaming potential (eq 6) and distribution of ζ-potential (eq 10) depend only on this combination. When parameter *A* is small, by developing eq 15 in Taylor series in *A*, we can see that Φ – *φ*(0) ≈ ζ_0_. By substituting this into eq 9 and developing the exponential function in series for small *A*, we obtain
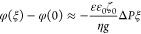
16which is the same behavior as in the classical case of dielectric substrates.

Above we have considered the simplest case of constant “chemical” charge density independent of ζ-potential. Given that this charge is a result of preferential ion adsorption or dissociation of ionogenic groups, it typically depends on the concentration of some ions at the surface. This, in turn, is affected by electrostatic attraction/repulsion. Therefore, generally, the density of “chemical” surface charge should be considered a function of ζ-potential, *σ*(ζ). In the case of electron-conducting substrates, this potential is controlled not only by the “chemical” charge but also by the electron/hole polarization charges. However, whatever the mechanism of “chemical”-charge formation, the total polarization charge must be zero for ungrounded conductors. Therefore, the right-hand side of eq 12 should still be equal to the total “chemical” charge. With a charge regulation, the latter becomes dependent on ζ-potential, which changes with coordinate according to eq 10. Hence, on the left-hand side of eq 12, we should average the “chemical” charge density over the capillary length to obtain
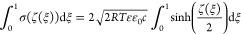
17

For the distribution of ζ-potential, we can still use eq 10, so
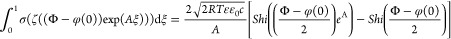
18where we have taken the integral from the right-hand side of eq 17. As previously, (Φ – *φ*(0)) can be found from eq 18 solved as a transcendental equation.

Within the scope of the popular charge regulation model,^19^ the surface charge is described by the so-called Langmuir–Stern isotherm, which gives
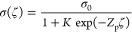
19where *Z*_p_ is the charge (in proton-charge units) of potential-determining ions and *σ*_0_ is the maximum surface-charge density corresponding to full dissociation. Constant *K* is proportional to the bulk concentration of potential-determining ions and, thus, is a function of solution pH, for example, in the case of weakly acidic groups. The term with the exponent in the denominator reflects the fact that the surface concentration of ions is different from their bulk concentration due to electrostatic repulsion/attraction. Thus, for instance, an increase in the negative surface-charge density with increasing pH is accompanied by the intensification of electrostatic attraction of H^+^ ions, which somewhat reduces the degree of dissociation.

Especially large values of dimensionless pressure differences can be expected in capillarity-driven electrokinetic phenomena, in particular, in systems with side evaporation from thin (nano)porous films (see Figure 4 for the schematic). A simple model for the distribution of hydrostatic pressure in such systems has recently been developed in ref (10) using the Darcy law for the description of viscous flow along the film and assuming a constant evaporation rate (controlled by the external mass transfer) from the fully wet part of the film. Under these assumptions, one obtains a linearly decreasing hydrostatic-pressure gradient along the film (in contrast to the constant pressure gradient occurring in the pressure-driven mode), which is because ever more liquid is lost to evaporation while moving along the film. The corresponding expression is
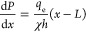
20where *P* is the hydrostatic pressure, *x* is the coordinate along the film, *h* is the film thickness, *L* is its length, *χ* is its hydraulic permeability, and *q*_e_ is the linear evaporation rate (m/s). The evaporation rate is assumed to be constant along the film (we disregard the dependence of saturated-vapor pressure on the menisci curvature). After integration along the film (taking into account that pressure at the immersed end equals atmospheric (zero relative) pressure), we obtain
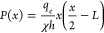
21

As discussed in ref (10), the tangential hydraulic flow is driven by the gradient of (negative) capillary pressure arising beneath the curved menisci at the external film surface. While moving along the film away from the immersed end, ever larger negative pressures are required to drive the viscous flow along the ever longer film segment. This negative-pressure buildup occurs due to a gradually increasing menisci curvature, which keeps growing until it reaches the maximum corresponding to the pore size. Once this state is reached, the menisci start to recede into the pores. Thus, the maximum pressure difference along the film is equal to the maximum negative capillary pressure. The length of the fully wet zone can be found by substituting negative maximum capillary pressure, −*P*_cm_, into eq 21
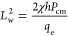
22

For the gradient of streaming potential, eq 1 is still applicable, though the hydrostatic-pressure gradient is not constant anymore but is given by eq 20 from which we obtain
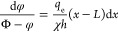
23

After integration
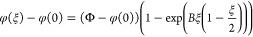
24where ξ ≡ *x*/*L* is the dimensionless coordinate along the porous film
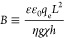
25

The local ζ-potential, ζ(*ξ*) ≡ Φ – φ(*ξ*), is given by
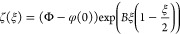
26

Taking into account as previously that the total induced electron/hole charge is zero and using eq 11, in the approximation of constant “chemical” charge, we obtain
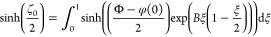
27

From this transcendental equation, one can find (Φ – *φ*(0)) as a function of *B*. From eq 24, we obtain this expression for the coordinate dependence of derivative of electrostatic potential
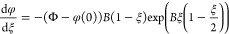
28

Using eqs 22 and 25, for the dimensionless parameter *B* occurring at the maximum wet length, *L*_w_, we get
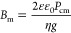
29

For the maximum capillary pressure, we have
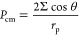
30where ∑ is the surface tension, *θ* is the contact angle, and *r*_p_ is the pore radius.

## Results and Discussion

**Figure 2:**
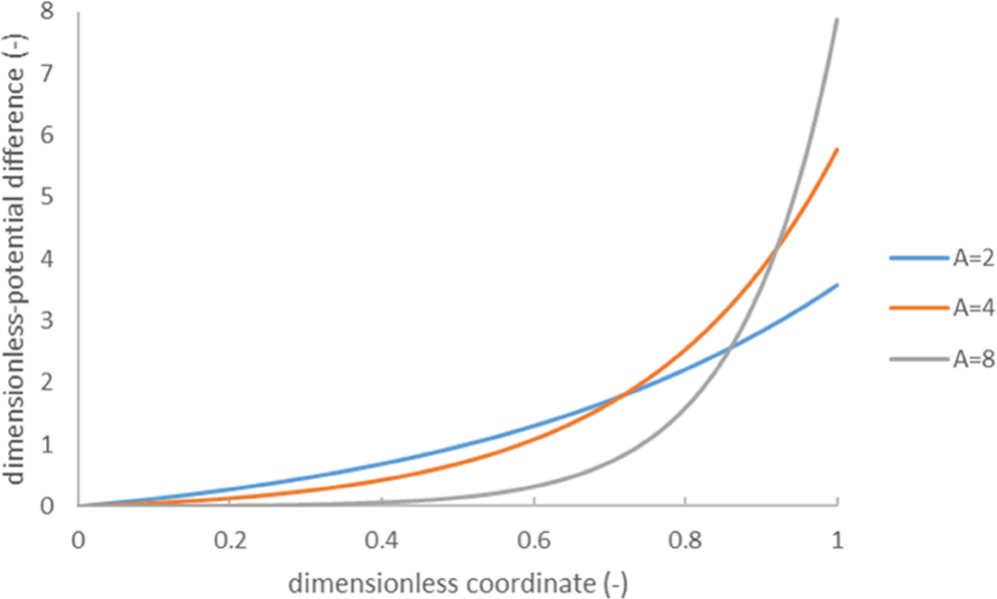
Distribution of dimensionless-potential difference along capillary, constant charge model, ζ_0_ = 2; the values of dimensionless pressure are indicated in the legend.

Taking into account that integral hyperbolic sinus is a strongly increasing function of its argument, eq 15 shows that when parameter *A* increases, (Φ – *φ*(0)) → 0. Physically, this means that the polarization charges distribute in such a way that the net surface-charge density (fixed plus induced charges) at the capillary “entrance” tends to zero, whereas it “peaks” exponentially ever stronger (with increasing pressure difference) close to the “exit” (see eq 10). This is illustrated in Figure 2.

**Figure 3:**
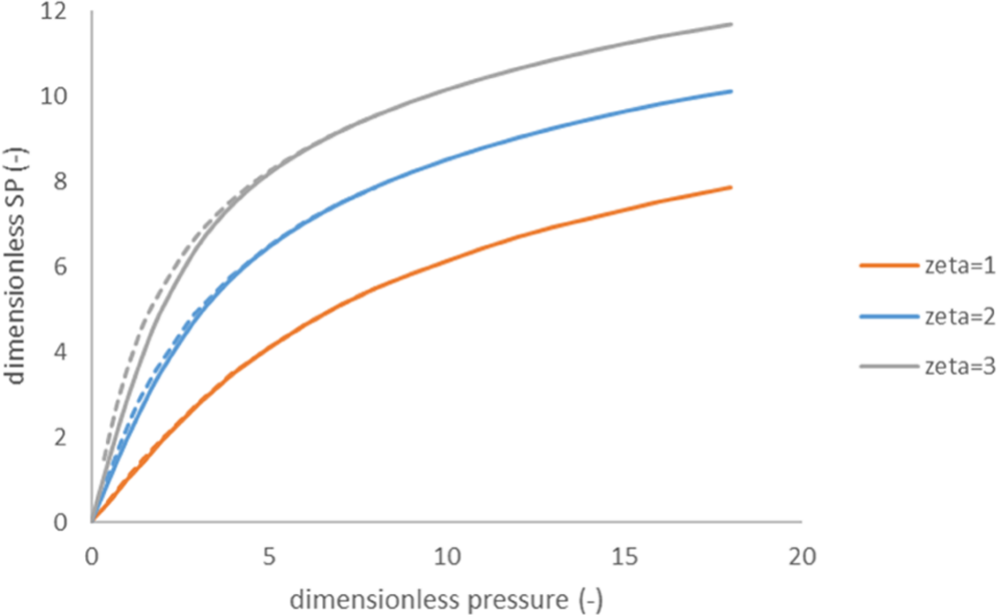
Dimensionless streaming potential as a function of dimensionless hydrostatic-pressure difference (parameter *A* defined by eq 7); the values of “equilibrium” dimensionless ζ-potential (ζ_0_) are indicated in the legend; the dashed lines show calculations with the approximate eq 31.

Figure 3 shows the dependence of streaming potential on the dimensionless pressure (parameter *A*). At its larger values, the dependence is essentially sublinear. At the same time, Figure 3 confirms that at small values of parameter *A*, the dependence is linear. We have just seen that this coincides with the classical Smoluchowski equation.

The numerical solution of eq 15 shows that while Φ – *φ*(0) → 0 at large *A*, , so the second term on the right-hand side of eq 15 can be neglected, and
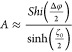
31

This inverse relationship between streaming potential and dimensionless pressure has an accuracy better than 1–2% at *A* > 5. At relatively small ζ-potentials (as long as ), it provides a good approximation at a small *A*, too. At larger ζ-potentials, one can combine the linear approximation Δφ = *A*ζ_0_ with eq 31, some deviations from either of them occurring only at intermediate values of *A*. Figure 3 confirms the good applicability of eq 31.

**Figure 4:**
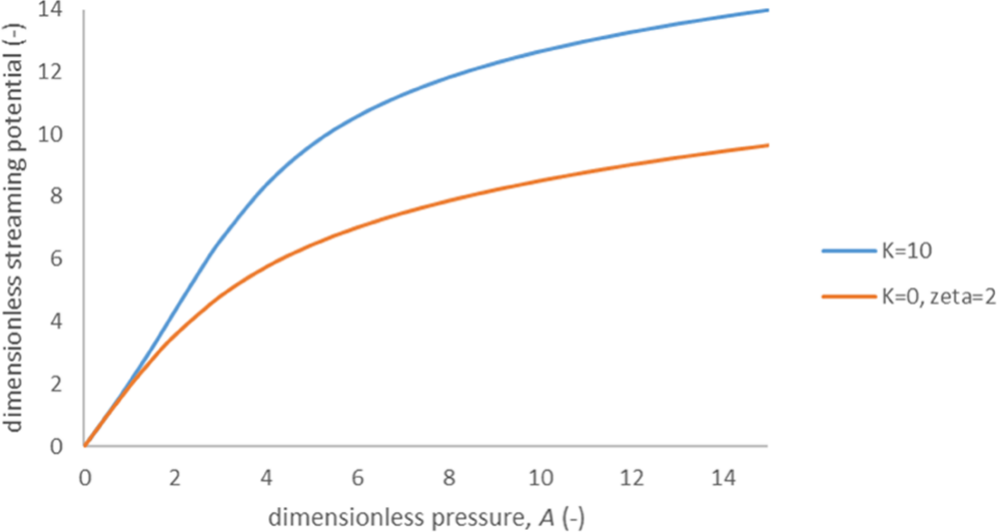
Dimensionless streaming potential vs dimensionless pressure for charge regulation (blue) and constant charge density (orange).

Figure 4 shows a comparison of pressure dependence of streaming potential calculated for the case of charge regulation using eqs 6 and 18 with the case of constant charge (eqs 6 and 15). The maximum surface-charge density in the case of charge regulation, *σ*_0_, is assumed to correspond to the same “zero-flow” dimensionless ζ-potential as in the case of constant charge.

As we can see, charge regulation can make the nonlinearity occur at somewhat larger dimensionless hydrostatic pressure differences, but qualitatively the behavior remains the same.

### Scenarios of Experimental Verification

#### Pressure-Driven Mode

Above, we have seen that the extent of the nonlinearity (which distinguishes SP with electron-conducting substrates from the classical case) is directly proportional to the hydrostatic pressure drop and inversely proportional to the solution conductivity. Using the model of identical straight parallel cylindrical capillaries, the pressure drop can be expressed this way
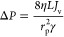
32where *J*_v_ is the volume flux (m/s), *r*_p_ is the pore radius, and γ is the porosity (for tortuous pores, it also includes a tortuosity factor). Accordingly (see eq 7)
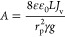
33

Figure 2 shows that the nonlinearity becomes noticeable when *A* ≥ 2 ÷ 3. This parameter gets larger, in particular, in solutions of lower electric conductivity. For our simple model to be applicable, the capillaries have to be sufficiently broad compared to the thickness of diffuse parts of EDLs. The latter is known to increase with decreasing electrolyte concentration (solution conductivity) inversely proportionally to the square root of it.^20^ Therefore, to maintain the impact of diffuse parts of EDLs at an acceptably low level, a decrease in concentration should be accompanied by an increase in the capillary radius. The latter implies less pressure drop at a given volume flux. As we can see from eq 33, parameter *A* is inversely proportional to the square of capillary radius. Therefore, at a given volume flux, reducing electrolyte concentration (and proportionally increasing the capillary radius) would leave parameter *A* (and the extent of nonlinearity) unchanged. At the same time, this would lead to an increase in Reynolds number and (in sufficiently broad capillaries) may result in deviations from the laminar flow pattern.^21^ Another way to increase the “effective pressure drop” is using thicker diaphragms with relatively small pores. Thus, for instance, assuming the thickness (capillary length) of *L* = 1 cm, the capillary radius of *r*_p_ = 0.5 μm, the active porosity of γ = 0.1, 1 mM NaCl solution, and a “reasonable” linear filtration rate of 0.3 mm/s, we obtain *A* ≈ 7. In 1 mM electrolyte solutions of (1:1) electrolytes, the EDL thickness is about 10 nm, which is around 50 times less than the assumed capillary radius (hence, no EDL overlap and surface conductance). Therefore, to achieve this flow rate in such a diaphragm, a pressure difference of about 1 MPa has to be applied. Sintered metals with average pore sizes down to single micrometers are commercially available.^22^ Nevertheless, exploration of pronounced nonlinearity requires other recipes.

#### Evaporation-Driven Mode

**Figure 5:**
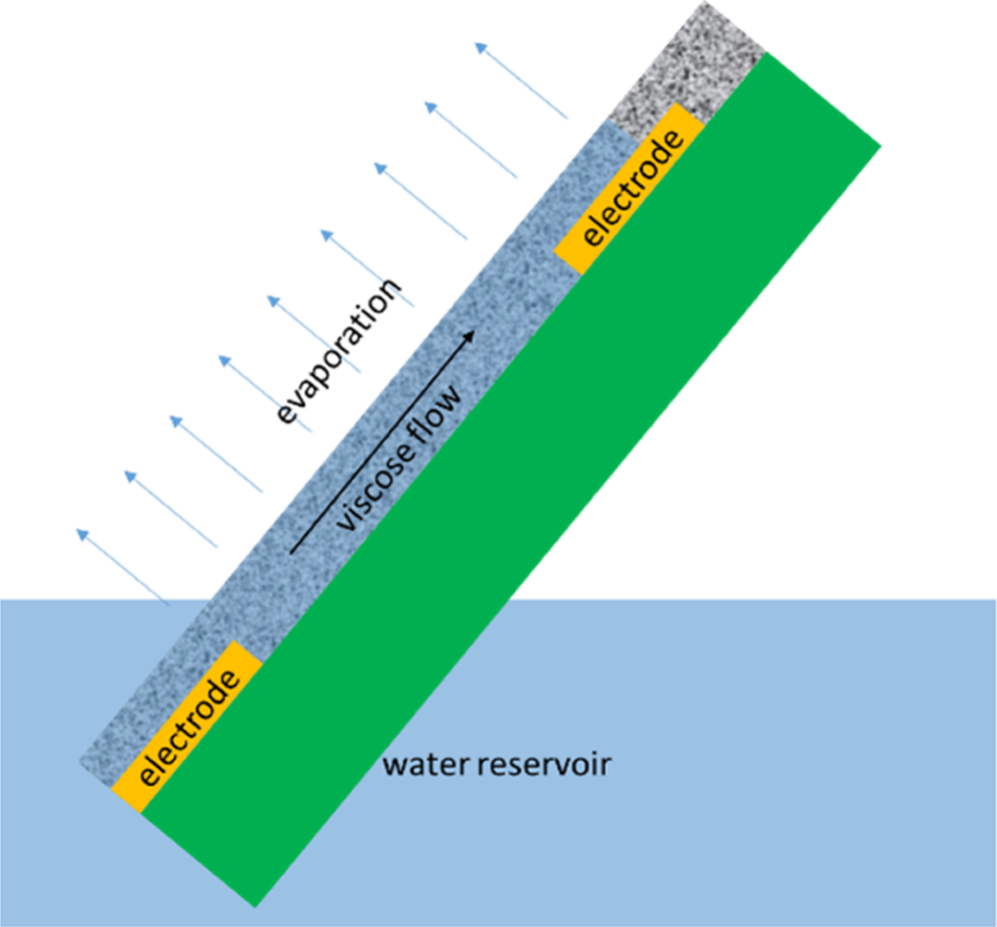
Schematic of systems with “side” evaporation (not to scale); green color shows a nonporous nonconducting substrate.

In hydrophilic nanopores, capillary pressures can be very high (>10 MPa). In this subsection, we will demonstrate that this can lead to very large pressure differences along thin nanoporous films under evaporation conditions. These, in turn, can give rise to large “dimensionless pressures”. In some studies, thin nanoporous films were assembled from electron-conducting nanoparticles (for example, carbon black).^7−9^ In a typical configuration (see Figure 5), a thin (supported or stand-alone) film of a nanoporous material is immersed with one extremity in an (aqueous) electrolyte solution. The liquid is sucked into the pores by capillary forces. Simultaneously, the solvent evaporates predominantly from the film side surface. There are, at least, two electrode stripes: one located close to the immersed film extremity and another situated at a certain distance along the film length (the direction of capillary imbibition). In some studies, there have been additional intermediate electrodes used to monitor the open-circuit voltage distribution along the film.

**Figure 6:**
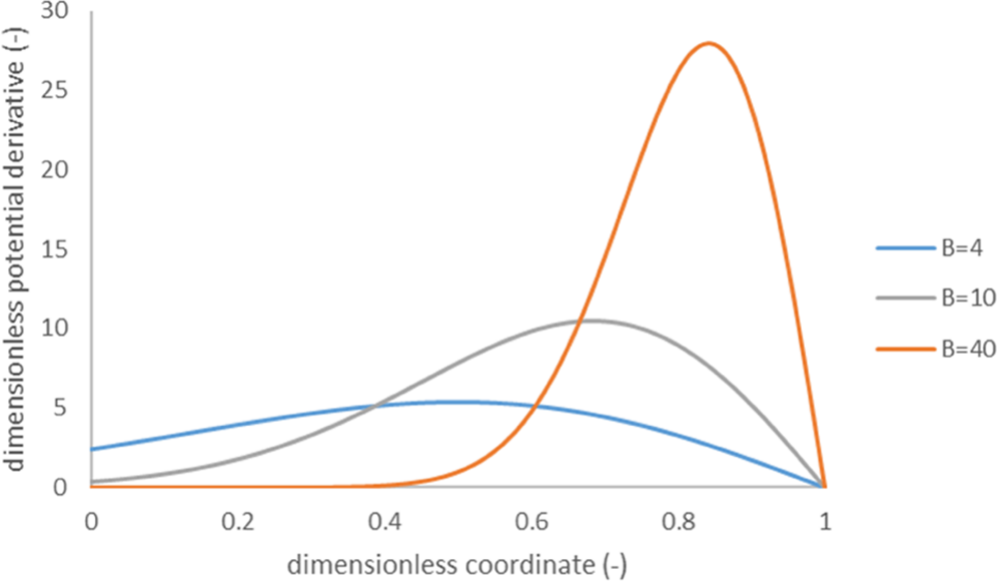
Distribution of derivative of dimensionless open-circuit voltage with respect to dimensionless coordinate along a porous film calculated using eqs 27 and 28: ζ_0_ = −3, the values of dimensionless parameter *B* are indicated in the legend.

Assuming as previously a 1 mM aqueous NaCl solution, the pore radius of 0.5 μm, and perfect wetting according to eq 29 (*θ* = 0), for the dimensionless parameter B occurring at the maximum wet length, we obtain *B*_m_ ≈ 4. In contrast to parameter *A* (see eq 33) controlling the nonlinearity in the pressure-driven mode, parameter *B* is inversely proportional to the first power of pore radius. Therefore, reducing electrolyte concentration (and conductivity) while increasing the pore size to keep the ratio of pore radius and screening length constant causes an increase in parameter *B*_m_ inversely proportional to the square root of concentration. Thus, with 0.01 mM NaCl solution (and 5 μm pore radius), *B*_m_ ≈ 40. Incidentally, experimental studies used very dilute electrolyte solutions although the pore size was essentially smaller than 10 μm. Figure 5 shows examples of distribution of electrostatic potential derivative along porous film with evaporation. This distribution is in good qualitative agreement with experimental data obtained in ref (8) for nanoporous films made from carbon black nanoparticles (see Figure 2 of ref (8)) (Figure 6).

## Conclusions

In the limiting case of sufficiently broad capillaries (“Smoluchowski limit”), streaming potential has long been considered to be a linear function of applied pressure. However, as we have demonstrated in this study, this generally applies only to nonconducting substrates. If substrates are electron-conducting (though still ideally polarizable, no electrode reactions), the linear behavior occurs only at sufficiently low dimensionless pressure differences directly proportional to hydrostatic pressure difference and inversely proportional to solution conductivity. At larger dimensionless pressure differences, the dependence becomes pronouncedly sublinear while the dependence on the coordinate along the flow direction is superlinear (exponential). The extent of nonlinearity also depends on the mechanism of surface-charge formation, charge regulation giving rise to a somewhat less pronounced nonlinearity. Experimental detection of predicted trends calls for the use of rather large applied pressures in systems with relatively large pores in dilute solutions. Alternatively, clear manifestations can be expected in devices where large hydrostatic-pressure differences are induced due to capillarity in water evaporation from nanoporous materials. Experimental data already published for such systems are in good qualitative agreement with the model predictions.
